# The Relationship Between the Virtual Hand Illusion and Motor Performance

**DOI:** 10.3389/fpsyg.2018.02242

**Published:** 2018-11-20

**Authors:** Satoshi Shibuya, Satoshi Unenaka, Yukari Ohki

**Affiliations:** ^1^Department of Integrative Physiology, Kyorin University School of Medicine, Tokyo, Japan; ^2^Department of Sport Education, School of Lifelong Sport, Hokusho University, Ebetsu, Japan

**Keywords:** bodily self-consciousness, agency, body ownership, movement error, rubber hand illusion, delayed visual feedback

## Abstract

Bodily self-consciousness consists of agency (i.e., the feeling of controlling one’s actions and causing external events) and body ownership (i.e., the feeling that one’s body belongs to one’s self). If a visual presentation of a virtual (fake) hand matches the active movement of a real hand, both the agency and body ownership of the virtual hand are induced [i.e., the active virtual hand illusion (VHI)]. However, previous active VHI studies have rarely considered the effects of goal-related movement errors (i.e., motor performance) on the senses of agency and ownership. Hence, the current study aimed to clarify the relationship between the active VHI and motor performance. To induce the VHI, 18 healthy subjects (three men and 15 women; 20.7 ± 7.3 years) were required to continuously move a virtual hand around a circle at a predetermined speed (i.e., spatial and temporal goals) using their active hand movements. While moving the virtual hand actively, five visual feedback delays were introduced: 90, 210, 330, 450, and 570 ms. It was found that the subjective ratings of both the agency and body ownership of the virtual hand decreased as a function of the delay intervals, whereas most of the spatial and temporal movement errors linearly increased. Using multiple regression analyses, we examined whether the agency and ownership ratings could be explained effectively by both the delay and movement errors. The results demonstrated that the agency was determined not only by the delay but also by the movement variability, whereas the body ownership was mostly determined by the delay. These findings suggest a possibility that the goal-related motor performance of the active VHI influences the agency judgment more strongly, while its effect on the ownership judgment is weaker.

## Introduction

Bodily self-consciousness comprises the senses of agency and body ownership (Gallagher, 2000, 2005). Agency refers to the subjective feeling of controlling one’s actions and causing external events, while body ownership refers to the feeling that one’s body (parts) belongs to one’s self. In the current study, for body ownership, we focus on the ownership of the hand, although many studies have investigated the self-attribution of different body parts, such as the trunk (Ehrsson, 2007; Lenggenhager et al., 2007), foot (Crea et al., 2015), or face (Tsakiris, 2008).

Regarding the sense of agency, its underlying mechanisms remain controversial. To explain the emergence of agency, earlier studies used a CM (Frith et al., 2000; Blakemore et al., 2002), which was originally developed as a motor control theory (Wolpert and Ghahramani, 2000). The CM proposes that the brain compares predicted sensory feedback based on an efference copy of motor signals with the actual sensory feedback, which we will refer to as the feedback comparison hereafter. If these two sources of feedback match, a sense of agency is induced. Likewise, a large discrepancy between the two reduces or eliminates the experience of agency. In contrast to the CM, a postdictive account of agency proposes that agency occurs due to a causal inference between an intention and an observed action via sensory inputs (i.e., external information) (Wegner, 2003; Wegner et al., 2004), which does not require internal prediction mechanisms that precede voluntary actions, as suggested by the CM. As a compromise between the CM and postdictive inference account, recent studies have proposed that agency results from the optimal integration of internal cues involving the feedback comparison and external cues (e.g., contextual information), which are weighted according to their reliability and availability in a given situation (Moore and Fletcher, 2012; Synofzik et al., 2013).

Hand ownership can be examined by using the RHI. In the original RHI (Botvinick and Cohen, 1998), an experimenter continuously strokes a subject’s visually occluded hand and a visible fake (rubber) hand in synchrony, inducing subjects to experience touch sensations where the fake hand is stroked; accordingly, there is a sense of illusory ownership of the fake hand. If the visuo-tactile stimulation between the fake and real hands occurs in asynchrony, the RHI is either reduced or abolished. This suggests that multisensory integrations (i.e., between vision, touch, and proprioception) are necessary to induce the visuo-tactile RHI (Makin et al., 2008; Tsakiris, 2010).

Recent studies have investigated both agency and hand ownership by using the active RHI (Tsakiris et al., 2006; Dummer et al., 2009; Kalckert and Ehrsson, 2012, 2014; Braun et al., 2014; Caspar et al., 2015; Hara et al., 2015; Ismail and Shimada, 2016; Salomon et al., 2016). That is, if the visual presentation of the fake (or virtual) hand movement matches the subject’s active hand movement, both the agency and ownership of the fake hand are induced at the same time. In most of the active RHI studies, a simple finger movement without a specific goal has been used to induce the illusion. For example, in the studies by Kalckert and Ehrsson (2012) and Braun et al. (2014), the subjects controlled the index finger of the fake hand by moving their invisible index finger (i.e., finger tapping). To control the virtual hand (fingers), some studies also required subjects to move their all digits at the same time (i.e., the movement of opening and closing the hand) (Ismail and Shimada, 2016) or in a series, such as when finger-counting (Sanchez-Vives et al., 2010).

Asynchronous movements of the fake and real hands reduce both the agency and body ownership in the active RHI. This suggests that the agency requires a match between the predicted and actual sensory feedback of the movement (i.e., feedback comparison), whereas the body ownership requires a temporal matching between different modalities (i.e., multisensory integration), though an interaction between the two is still controversial. However, prior studies of the active RHI also reported that the sense of ownership breaks down completely in the asynchronous condition (circa a 500 ms delay between the fake and real hand movements), while the sense of agency slightly remains (e.g., Kalckert and Ehrsson, 2012; Ismail and Shimada, 2016). These findings might support the possibility that the agency is determined not only by the feedback comparison but also by other internal (e.g., motor intention) and external cues (e.g., motor outcome), whereas the body ownership mainly occurs due to the multisensory integration.

As the previous active RHI studies have used simple hand (finger) movements without a specific goal, no study has investigated how movement errors (i.e., the accuracy and variability of the fake hand movement relative to the goal) influence the agency and body ownership when using the active RHI design. Therefore, the current study aimed to examine the effects of goal-related movement errors (i.e., motor outcome) on the senses of agency and ownership. In the current experiment, we required subjects to repeatedly move a virtual hand around a circle at a predetermined speed (i.e., spatial and temporal goals) using their active hand movements (i.e., the active VHI). To vary the difficulty of the feedback comparison (agency) and multisensory integration (ownership), we used five visual feedback delays while moving the virtual hand: 90, 210, 330, 450, and 570 ms. We predicted that both the agency and ownership of the virtual hand, as assessed by questionnaire ratings, would be impaired as a function of the delay intervals (Ismail and Shimada, 2016), while the goal-related movement errors would gradually increase (Smith et al., 1960). Using multiple regression analyses, we tested whether the subjective ratings of the agency and body ownership could be explained effectively by a combination of the visual feedback delay and movement errors.

According to the optimal cue integration account (Moore and Fletcher, 2012; Synofzik et al., 2013), we hypothesized that the agency in the active RHI would be determined by both the delay (i.e., feedback comparison) and the movement errors as the external cue. On the other hand, we also hypothesized that the body ownership would be mostly determined by the delay, based on the predominance of the multisensory integrations. Consequently, we predicted that the motor performance of the active VHI would influence the agency judgment more strongly, while its effect on the ownership judgment would be smaller.

## Materials and Methods

### Subjects

Eighteen healthy subjects (three men and 15 women; age range 18–49 years; median age 18.5 years) participated in this study. The subjects were blinded to the purpose of the experiment, and all were right-handed according to the Edinburgh Inventory (Oldfield, 1971). This study was approved by the institutional review board at the Kyorin University School of Medicine and conducted according to the principles and guidelines of the Declaration of Helsinki. All subjects provided written informed consent prior to the study in accordance with the institutional guidelines.

### Apparatus

**FIGURE 1:**
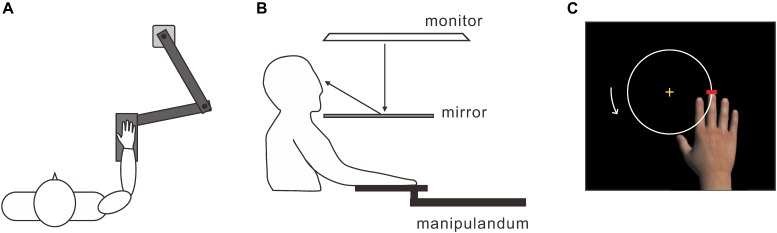
Experimental apparatus. **(A)** A horizontal planar manipulandum with a two-joint mechanical arm for measuring the position of the subject’s right hand. Subjects put their right hand on a support that was attached to one end of the arm. **(B)** Visual images projected onto the monitor were displayed on the horizontal planar workspace via a half-silvered mirror. **(C)** To induce the virtual hand illusion, subjects moved the tip of the middle finger of the virtual hand counterclockwise around a white circle using an active hand movement.

To measure the subject’s hand movements, we used a horizontal planar manipulandum with a two-joint mechanical arm (MP-201P, Uchida Electronics Co., Tokyo, Japan) (Figure 1A). A support plate (29 × 11 cm) was attached at one end of the mechanical arm, whose position was measured at 100 Hz. The subjects were seated, and they placed their right hand palm down on the support. The tip of the subject’s middle finger corresponded to the distal end of the mechanical arm. A 46-inch monitor (LB-T461, Sharp, Tokyo, Japan) was placed 60 cm above the horizontal planar workspace with the screen facing down (Figure 1B). A half-silvered mirror was positioned horizontally 30 cm above the workspace, so that the visual images on the monitor were displayed on the workspace. This setup prevented a direct view of the subject’s hand. The positional data were acquired by two laboratory computers: one for analyzing the data offline and the other for controlling the visual hand on the monitor. The delivery of the visual images and the experimental timing were controlled using Presentation version 16.0 (Neurobehavioral Systems Inc., Berkeley, CA, United States). The inherent delay was approximately 90 ms, which is below the threshold for detecting a visual feedback delay (i.e., the 90 ms-delay condition) (Shimada et al., 2010). We also introduced four artificial delays of 120, 240, 360, and 480 ms using a hardware device (UDD-30-2, Uchida Electronics Co., Tokyo, Japan), resulting in actual time delays of 210, 330, 450, and 570 ms. Accordingly, there were five delay conditions from 90 to 570 ms at intervals of 120 ms.

### Procedure

Each subject performed the five delay conditions in a pseudo-random order. Each condition started with the presentation of a life-sized virtual image of a right hand with its palm down, a white circle that was 10 cm in diameter, and a short red line to designate the home position (Figure 1C). The virtual hand could be moved by the subject’s active hand movement. In the 90 ms-delay condition (i.e., in which there was no artificial delay), the virtual hand was always displayed in front of the real one so that the distance between the middle finger tips was 12 cm. However, this spatial arrangement gradually deteriorated as the artificial delays increased. After holding the middle finger of the virtual hand at the home position for 3 s, the color of the line altered from red to green, serving as the start signal. Subjects were required to move the virtual middle fingertip counterclockwise around the circle for 2 min. To keep the MT as constant as possible, periodic pure tones were delivered once every 3.5 s during the movement. Subjects made one circular movement every 3.5 s, as accurately as possible. After 2 min, the color of the short line returned from green to red, signaling the completion of the circular movements, and subjects were required to return the virtual middle fingertip to the home position.

After each condition, subjects were allowed to have a 10-min rest before the next condition. During this period, they reported their subjective experiences during moving the virtual hand using a questionnaire with a 7-point Likert scale, ranging from +3 (agree strongly) to -3 (disagree strongly). This questionnaire consisted of eight items, based on a previous study (Kalckert and Ehrsson, 2012) (Table 1). These items were divided into the following four categories: *ownership* (Q1 and Q2), *ownership control* (Q3 and Q4), *agency* (Q5 and Q6), and *agency control* (Q7 and Q8). According to previous active RHI studies (Kalckert and Ehrsson, 2012; Braun et al., 2014), the averaged ratings within each category were used for the statistical analyses.

**Table 1:** The eight items and four categories of the questionnaire that evaluated the subjective experiences of agency and ownership.

Category	Question
Ownership	1. It seemed as if I were sensing the movement of my hand in the location where the virtual hand moved.
	2. I felt as if the virtual hand was part of my body.
Ownership control	3. It felt as if I no longer had a right hand and as if my right hand had disappeared.
	4. It seemed that I had two right hands.
Agency	5. The virtual hand moved just like I wanted it to, as if it was obeying my will.
	6. I felt as if I was controlling the movements of the virtual hand.
Agency control	7. I felt as if the virtual hand was controlling my movements.
	8. It seemed that the virtual hand had a will of its own.


### Data Analysis of the Circular Movements

**FIGURE 2:**
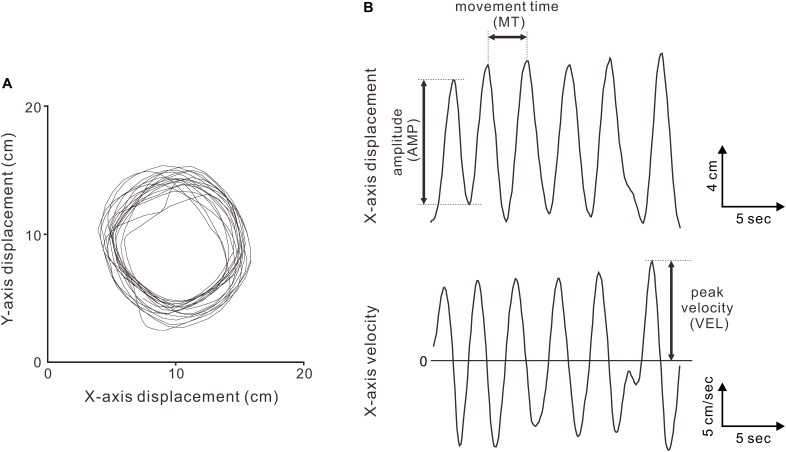
Kinematics of the circular movements. **(A)** Displacement data (*x*-*y* plot) for two minutes in a representative subject (the 90 ms-delay condition). **(B)** Displacement (top) and velocity (bottom) data in the *x*-axis dimension. The peak-to-peak amplitude (AMP), movement time per cycle (MT), and peak velocity (VEL) were measured.

The circular movements were analyzed using custom scripts in MATLAB, version 8.1 (The MathWorks Inc., Natick, MA, United States). Based on a previous study (Franz, 2003), the displacement data of the circular movements in each condition (Figure 2A) were deconstructed into *x*- and *y*-axis displacements, resulting in two sets of semi-sinusoidal data (Figure 2B, top). In addition, the velocity data for the *x*- and *y*-axis dimensions were obtained as functions of time using numerical time differentiation (Figure 2B, bottom). For the indices of the movement accuracy, we extracted the AMP and MT per cycle from the displacement data and extracted the VEL from the velocity data. Regarding the indices of the movement variability, we calculated the VE of the AMPs (VE-AMP), MTs (VE-MT), and VELs (VE-VEL).

### Statistical Analysis

All statistical analyses were performed using the R statistical software, version 3.2.3 (R Foundation for Statistical Computing, Vienna, Austria), and the level of probability for statistical significance was *p* < 0.05.

## Results

### Subjective Ratings

**FIGURE 3:**
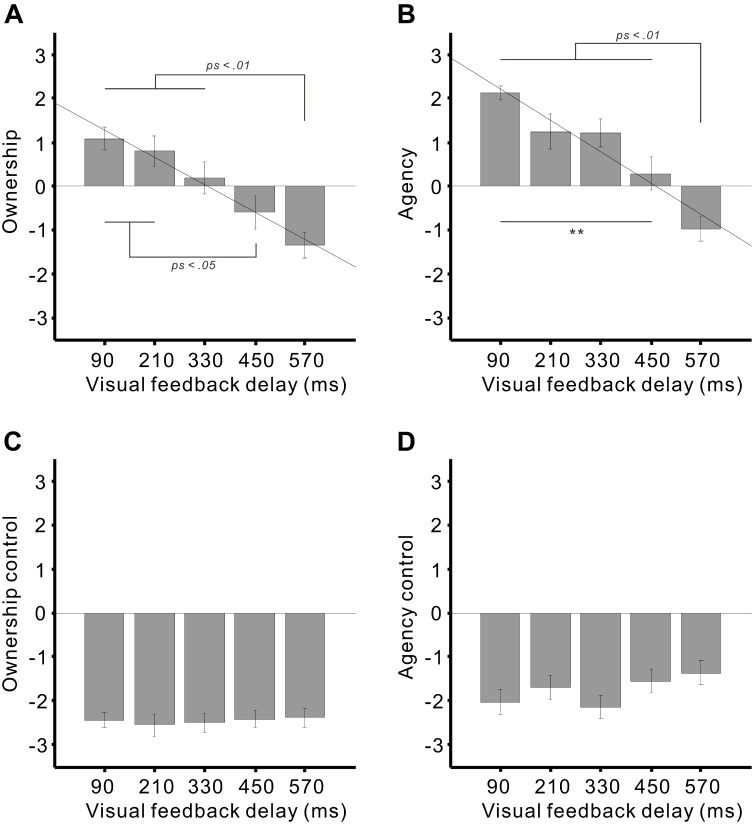
Mean subjective ratings of **(A)** ownership, **(B)** agency, **(C)** ownership control, and **(D)** agency control against the visual feedback delays. Subjects rated their subjective feelings while moving the virtual hand, using a 7-point Likert scale, ranging from +3 (agree strongly) to –3 (disagree strongly). Diagonal lines denote the regression lines. Error bars represent ± 1.0 standard error. ^∗∗^*p* < 0.01.

A one-sample Kolmogorov–Smirnov goodness-of-fit test confirmed that the subjective ratings had a normal distribution. We applied a one-way analysis of variance to the ratings for each category. As a result, significant main effects of the delay were found in the ratings for *ownership* (*F*_4,68_ = 11.3, *p* < 0.01; η^2^ = 0.4; Figure 3A) and *agency* (*F*_4,68_ = 21.1, *p* < 0.01; η^2^ = 0.55; Figure 3B) but not for *ownership control* (*p* > 0.9; Figure 3C) or *agency control* (*p* > 0.1; Figure 3D).

With regard to the ownership rating, positive ratings for the 90 and 210 ms delays were significantly higher than the negative ratings for the 450 ms (both *p* < 0.05) and 570 ms delays (both *p* < 0.01) (Tukey’s honest significant difference [HSD] test). A significant difference was also detected between the 330 and 570 ms delays (*p* < 0.01). A simple linear regression analysis revealed that the visual feedback delay significantly explained the ownership rating (adjusted *R*^2^ = 0.28, β = –0.54, *t*_88_ = –5.98, *p* < 0.01).

For the agency rating, positive ratings for the delays of 90–450 ms were significantly greater than the negative ratings for the 570 ms delay (all *ps* < 0.01). A significant difference was also identified between the 90 and 450 ms delays (*p* < 0.01). A regression analysis demonstrated that the delay significantly explained the agency rating (adjusted *R*^2^ = 0.35, β = –0.60, *t*_88_ = –7.06, *p* < 0.01).

We further tested whether positive responses (i.e., affirmation) of the ownership and agency were statistically higher than zero (neutral) using a one-sample *t*-test. The ownership ratings were significantly greater than zero in the 90 ms (*t*_17_ = 4.22, *p* < 0.01) and 210 ms delays (*t*_17_ = 2.31, *p* < 0.05) but not in the 330 ms delay (*p* > 0.6). On the other hand, the agency ratings were significantly higher than zero in the 90 ms (*t*_17_ = 12.8, *p* < 0.01), 210 ms (*t*_17_ = 3.17, *p* < 0.01), and 330 ms delays (*t*_17_ = 3.86, *p* < 0.01) but not in the 450 ms delay (*p* > 0.4).

### Performance of the Circular Movements

To identify the characteristics of the circular movements, we first examined the difference between the *x*- and *y*-axis data across the delay for each movement parameter using the paired *t*-test (Table 2). In all parameters, except for the VE-MT (*p* > 0.9), significant differences were detected between the two (all *ps* < 0.01). The *x*-axis displacement was greater (the AMP) and more variable (the VE-AMP) than the *y*-axis displacement. Additionally, the *x*-axis velocity was higher and more variable compared to the *y*-axis velocity. Correlation analyses revealed that the *x*- and *y*-axis data were highly correlated in all parameters (*r* = 0.78–0.99, all *ps* < 0.01).

**Table 2:** The means and standard deviations of the *x*- and *y*-axis dimensions and statistical significance between the two for all movement parameters.

	*x*-axis	*y*-axis	*t-*value	correlation coefficient
AMP (cm)	12.3 (0.7)	11.8 (0.8)	21.9^∗∗^	0.78^∗∗^
VE-AMP (cm)	1.46 (0.32)	1.20 (0.22)	38.2^∗∗^	0.85^∗∗^
MT (s)	3.34 (0.22)	3.35 (0.21)	10.1^∗∗^	0.99^∗∗^
VE-MT (s)	0.35 (0.06)	0.35 (0.05)	0.01	0.94^∗∗^
VEL (cm/s)	12.6 (1.3)	11.1 (1.3)	123.9^∗∗^	0.90^∗∗^
VE-VEL (cm/s)	2.3 (0.6)	2.1 (0.4)	9.66^∗∗^	0.84^∗∗^


*^∗∗^*p* < 0.01. AMP, amplitude; VE-AMP, variable error of the AMP; MT, movement time; VE-MT, variable error of the MT; VEL, velocity; VE-VEL, variable error of the VEL. Values in parentheses denote the standard deviation. The *t*-values were calculated using the paired *t*-test to compare the *x*- and *y*-axis data. Pearson’s correlation coefficients between the *x*- and *y*-axis data were computed.*

**FIGURE 4:**
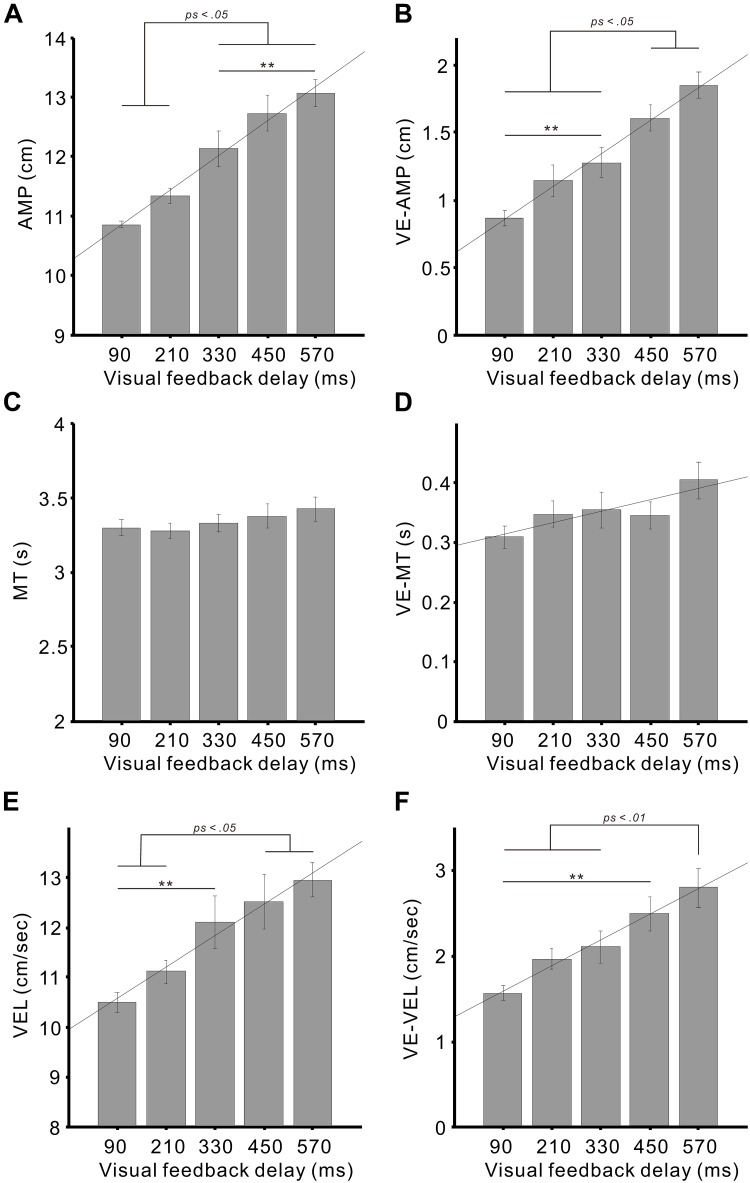
Mean movement errors of the circular movements against the visual feedback delays. **(A)** Amplitude (AMP), **(B)** variable errors of the amplitude (VE-AMP), **(C)** movement time (MT), **(D)** variable errors of the movement time (VE-MT), **(E)** velocity (VEL), and **(F)** variable errors of the velocity (VE-VEL). Diagonal lines denote regression lines. Error bars represent ± 1.0 standard error. ^∗∗^*p* < 0.01.

Next, we investigated the effects of the delay intervals on the movement parameters using the combined *x*- and *y*-axis data. The one-way analysis of variance revealed significant main effects of the delay on both the AMP (*F*_4,68_ = 27.9, *p* < 0.01; η^2^ = 0.62; Figure 4A) and VE-AMP (*F*_4,68_ = 26.5, *p* < 0.01; η^2^ = 0.61; Figure 4B). Subsequent analyses showed that the AMPs for the delays of 330–570 ms were larger than those for the 90 and 210 ms delays (all *ps* < 0.05; Tukey’s HSD test; Figure 4A). The difference between the 330 and 570 ms delays was also significant (*p* < 0.01).

The VE-AMPs for the 450 and 570 ms delays were larger than for the delays of 90–330 ms (all *ps* < 0.05; Figure 4B). A significant difference was also detected between the 90 and 330 ms delays (*p* < 0.01). Simple linear regression analyses revealed that the delay significantly explained both the AMP (adjusted *R*^2^ = 0.43, β = 0.66, *t*_88_ = 8.22, *p* < 0.01) and VE-AMP (adjusted *R*^2^ = 0.41, β = 0.64, *t*_88_ = 7.87, *p* < 0.01).

With regard to the MT and VE-MT, the one-way analysis of variance revealed that there was no main effect on both the MT (*p* > 0.1; Figure 4C) and VE-MT (*p* > 0.1; Figure 4D). However, the regression analyses demonstrated that the delay significantly explained the VE-MT (adjusted *R*^2^ = 0.05, β = 0.24, *t*_88_ = 2.37, *p* < 0.05) but not the MT (*p* > 0.09).

With regard to the VEL, the one-way analysis of variance revealed significant main effects of the delay on both the VEL (*F*_4,68_ = 11.7, *p* < 0.01; η^2^ = 0.41; Figure 4E) and VE-VEL (*F*_4,68_ = 11.1, *p* < 0.01; η^2^ = 0.39; Figure 4F). The VELs for the 450 and 570 ms delays were significantly greater than for the 90 and 210 ms delays (all *ps* < 0.05). The difference between the 90 and 330 ms delays was also significant (*p* < 0.01).

The VE-VEL for the 570 ms delay was larger than for the 90–330 ms delays (all *ps* < 0.01). A significant difference was also detected between the 90 and 450 ms delays (*p* < 0.01). The simple regression analysis showed that the delay significantly explained both the VEL (adjusted *R*^2^ = 0.22, β = 0.48, *t*_88_ = 5.1, *p* < 0.01) and VE-VEL (adjusted *R*^2^ = 0.25, β = 0.51, *t*_88_ = 5.52, *p* < 0.01).

### Correlation and Regression Analyses

We also computed Pearson’s product-moment correlations to understand the relationships between all of the variables. Table 3 shows the correlation coefficients and their statistical significance. The agency and ownership ratings significantly correlated with the delay (*r* = -0.60 and -0.54) as well as the five movement parameters (all *ps* < 0.01; agency: *r* = -0.44 to -0.58; ownership: *r* = -0.28 to -0.39), but not with the MT. Regarding the movement parameters, stronger positive correlations were found between the amplitude and velocity (e.g., the AMP and VEL: *r* = 0.79; the VE-AMP and VE-VEL: *r* = 0.83).

**Table 3:** Correlation coefficients between all the parameters.

	2	3	4	5	6	7	8	9
1. Delay	–0.54**	–0.60**	0.66**	0.64**	0.17	0.25*	0.48**	0.51**
2. Ownership		0.61**	–0.33**	–0.39**	–0.12	–0.29**	–0.28**	–0.32**
3. Agency			–0.45**	–0.58**	–0.14	–0.44**	–0.39**	–0.56**
4. AMP				0.64**	–0.02	0.07	0.79**	0.58**
5. VE-AMP					–0.23*	0.43**	0.75**	0.83**
6. MT						0.27**	–0.53**	–0.31**
7. VE-MT							0.11	0.50**
8. VEL								0.79**
9. VE-VEL								


*^∗^p < 0.05, ^∗∗^*p* < 0.01. AMP, amplitude; VE-AMP, variable error of the AMP; MT, movement time; VE-MT, variable error of the MT; VEL, velocity; VE-VEL, variable error of the VEL.*

Accordingly, we further examined whether the agency and ownership ratings could be explained better by combining the delay and movement parameters than by the delay alone, as mentioned above. As such, a multiple regression analysis was conducted. To select an appropriate regression model, the explanatory variables were selected using the forward-stepwise method, based on Akaike’s information criterion (AIC) (Akaike, 1974) (i.e., the model with the lowest AIC value was selected). As a result, a significant regression model using the delay and movement parameters was found for both the agency (model: *y* = -0.003*x_1_* + -9.5*x_2_* + -0.14*x_3_* + 8.5, *x_1_* = delay, *x_2_* = VE-VEL, *x_3_* = MT; adjusted *R*^2^ = 0.47, *F*_3,86_ = 27.7, *p* < 0.01) and ownership ratings (model: *y* = -0.005*x_1_* + -0.26*x_2_* + 2.5, *x_1_* = delay, *x_2_* = VE-MT; adjusted *R*^2^ = 0.30, *F*_2,87_ = 20.2, *p* < 0.01). There were no obvious multicollinearities among the explanatory variables (variance inflation factors: <1.4). For the agency rating, all explanatory variables were statistically significant (delay: β = -0.32, *p* < 0.01; VE-VEL: β = -0.47, *p* < 0.001; MT: β = -0.23, *p* < 0.05). However, for the ownership rating, the delay (β = -0.50, *p* < 0.001) was significant, while the VE-MT (β = -0.17, *p* = 0.06) did not approach the significance level.

## Discussion

In the current study, we investigated the relationships between the active VHI and motor performance using goal-directed circular movements and delayed visual feedback. While the senses of agency and ownership of the virtual hand were impaired as a function of the visual feedback delay, the spatial and temporal errors of the circular movements linearly increased, except for the MT. The multiple regression analyses revealed that the agency could be explained well by both the delay and movement variability, whereas the body ownership was mostly explained by the delay, supporting our hypothesis. These results suggest that the motor performance of the active VHI can affect the judgment of agency more strongly, but that its effect on the ownership judgment is smaller.

Although both the ownership and agency ratings gradually decreased as a function of the visual feedback delays, a time window difference of their emergence was found. That is, the illusory ownership of the virtual hand was induced when the delay intervals were less than circa 300 ms (i.e., the 90 and 210 ms delays), whereas the agency was moderately experienced even when the delay was 330 ms. These findings are basically consistent with previous RHI (or VHI) studies that found that the ownership illusion is attenuated if the visuo-tactile or visuo-motor asynchrony during the induction of the illusion exceeds 200–300 ms (Shimada et al., 2009, 2014; Ismail and Shimada, 2016), while the sense of agency remains somewhat even when the visuo-motor asynchrony is around 500 ms in the active RHI setup (Kalckert and Ehrsson, 2012; Ismail and Shimada, 2016). Given our findings and the previous findings, the present results might indicate that body ownership emerges through a temporal binding (integration) of different sensory modalities, whereas agency is determined not only by a match between the predicted sensory feedback and actual feedback but also by other internal (e.g., motor intention) and external information (e.g., motor outcome).

The movement errors, except for the MT, revealed linear increases as a function of the delay intervals. Especially, the visual feedback delay correlated with the AMP (*r* = 0.66, *p* < 0.01) and VE-AMP (*r* = 0.64, *p* < 0.01) as spatial indices more strongly than the VE-MT (*r* = 0.25, *p* < 0.05) did as a temporal index. Moreover, a large increase in the rate from the 90 to 570 ms delays was observed in the VE-AMP (211%; [1.85/0.87 cm]) and VE-VEL (179%; [2.80/1.56 cm/s]) compared to the AMP (120%; [13.1/10.9 cm]), VEL (123%; [12.9/10.5 cm/s]), and VE-MT (129%; [0.40/0.31 s]), indicating that the spatial variability and velocity variability of the circular movements were more susceptible (or sensitive) to the visual feedback delay. Accordingly, it is natural that the subjects explicitly recognized their motor performance impairment, especially the increased movement variability, under the longer delay conditions.

The multiple regression analyses revealed that the agency ratings could be determined by both the delay and the movement parameters (i.e., the VE-VEL and MT). Especially, the effect of the VE-VEL (β = -0.47, *p* < 0.001) on the agency was greater than the effect of the delay (β = -0.32, *p* < 0.01) and MT (β = -0.23, *p* < 0.05). As such, when the multiple regression model added the delay to the two movement parameters (adjusted *R*^2^ = 0.47), it explained the agency rating better than the simple regression model using the delay alone (adjusted *R*^2^ = 0.35). On the other hand, the ownership rating was mainly caused by the delay (β = -0.50, *p* < 0.001), since the effect of the selected VE-MT (β = -0.17, *p* = 0.06) was weaker. Accordingly, the difference in the coefficients of determination between the multiple (adjusted *R*^2^ = 0.30) and simple regression models (adjusted *R*^2^ = 0.28) for the ownership rating was also smaller compared to the agency rating. These findings suggest that the goal-related movement variability in the active VHI design selectively functioned to determine the sense of agency. Our results may be partly explained by a recent optimal cue integration account that elucidates the experience of agency (Moore and Fletcher, 2012; Synofzik et al., 2013). This model proposes that agency is determined by the integration of internal (e.g., feedback comparison, knowledge, and belief) and external cues (e.g., contextual information), which are weighted depending on their reliability and availability. For instance, if a comparison between the predicted sensory feedback based on an efference copy and the actual feedback is unavailable or unreliable under a given condition, the brain takes account of other external agency cues, such as the visual consequence of the movement (Synofzik et al., 2010). In our experiment, the difficulty of the predicted/actual sensory feedback comparison would increase as the delay intervals increase. Additionally, the continuous circular movements would make the comparison more difficult compared to a discrete and single movement (Wen et al., 2015). Therefore, we infer that subjects would depend heavily on motor performance as an external cue for judging the agency.

Unlike finger movements in the previous active RHI studies, the current experiment used the arm movement to induce the illusory ownership of the virtual hand. When introducing the delayed visual feedback, this method produces a large spatial conflict between the seen and felt hand location, in addition to the temporal mismatch between different modalities. This inter-sensory conflict would be detected more easily as the delay intervals increase, and then would become a strong cue for the ownership judgment. Therefore, the subjects in this experiment may judge the ownership according to the inter-sensory conflict associated with the delay, rather than the movement performance (i.e., motor outcome).

There are at least two limitations in our study. One is that the weak effects of the motor performance on the ownership rating might be due to a lack of statistical power, since the sample size (*n* = 18) was relatively small. Therefore, future studies will need to increase the number of subjects to corroborate our findings more strongly. The other limitation is that we were unable to comment on the interaction between the agency and body ownership. However, previous studies comparing active and passive movements in the active RHI design have shown conflicting results. That is, some studies have reported that the ownership illusion is enhanced by active movements (agency) (Dummer et al., 2009; Kalckert and Ehrsson, 2012; Braun et al., 2014), whereas others have found no differences between the two (Walsh et al., 2011; Shibuya et al., 2017). As our correlation analyses identified a moderate relationship between the agency and ownership ratings (*r* = 0.61, *p* < 0.01), the agency might contribute to eliciting body ownership in the current experiment. However, we obviously need to conduct further elaborate experiments in the future to resolve this question. An interesting possibility might be to examine the sense of ownership by inducing the movement errors without any changes in the sense of agency (e.g., a pursuit task with the virtual hand).

## Author Contributions

SS, SU, and YO conceived and designed the experiment and discussed the results. SS performed the experiment, analyzed the data, and wrote the manuscript. All authors approved the final version of the manuscript.

## Conflict of Interest Statement

The authors declare that the research was conducted in the absence of any commercial or financial relationships that could be construed as a potential conflict of interest.

## Abbreviations

AMP: peak-to-peak amplitude
CM: comparator model
MT: movement time
RHI: rubber hand illusion
VE: variable errors
VE-AMP: variable error of the AMP
VEL: peak velocity
VE-MT: variable error of the MT
VE-VEL: variable error of the VEL
VHI: virtual hand illusion
